# British Medical Association

**Published:** 1903-08-01

**Authors:** 


### BRITISH MEDICAL ASSOCIATION.

### SEVENTY-FIRST ANNUAL MEETING AT SWANSEA.

Judging from the number of excursions arranged for and the length of the list of places of interest quoted in the *Daily Journal*, the 71st annual meeting of the British Medical Association, which commenced at Swansea on Tuesday, July 28th, should prove an interesting one, apart from the unique opportunity of studying the question of the housing of the working classes which this year's meeting affords. The proceedings of the Association were commenced by a meeting of the Council, followed by a service at St. Mary's Church, conducted by the Right Rev. the Lord Bishop of St. David's. The first general meeting of members was held at 2 P.M. at the King's Hall, the chair being taken by the President, Mr. Walter Whitehead. The minutes of the last annual meeting having been taken as read, Mr. Whitehead, after a few introductory words, vacated the chair in favour of the President for 1903–4, Dr. Thomas D. Griffiths, who, having been decorated with his badge of office, moved that H.R.H. the Prince of Wales be elected an honorary member of the British Medical Association. This resolution having been carried unanimously, and "God Save the Prince of Wales" having been sung, Dr. Ferguson moved a vote of thanks to Mr. Walter Whitehead, of whose services to surgery he spoke in the highest terms. This was seconded and carried with musical honours. Mr. Whitehead having responded, the chair was vacated by Dr. Griffiths in favour of the Chairman of the Council, who called upon the Treasurer to read the financial statement for the past year. A discussion thereon was opened by Dr. Bell, of Glasgow, who strongly criticised the management of the *British Medical Journal*, which he represented as uninteresting, and largely made up of ambiguous articles. A point of order having been raised and disallowed, Sir Victor Horsley spoke upon the subject, after which the discussion became general. It was finally proposed by the Treasurer that the accounts be passed as read, which was seconded and carried. Some further discussion took place upon the next motion, which was that a sum of £300 be voted at the next annual meeting to be added to the Superannuation Fund, during which it transpired that the basis of the Superannuation Fund, which was intended to benefit the clerical staff, was the Hastings Memorial Fund, after which, the auditors for the next year having been appointed, the meeting adjourned until 8 P.M., when the foreign and colonial guests were received by the President at the Albert Hall.

